# Effect of non-repetitive linker on in vitro and in vivo properties of an anti-VEGF scFv

**DOI:** 10.1038/s41598-022-09324-4

**Published:** 2022-03-31

**Authors:** Merve Arslan, Murat Karadag, Ebru Onal, Emine Gelinci, Gulcin Cakan-Akdogan, Sibel Kalyoncu

**Affiliations:** 1 Izmir Biomedicine and Genome Center, Izmir, Turkey; 2 Izmir International Biomedicine and Genome Institute, Dokuz Eylul University, Izmir, Turkey; 3 Institute of Health Sciences, Dokuz Eylul University, Izmir, Turkey; 4 Department of Medical Biology, Faculty of Medicine, Dokuz Eylul University, Izmir, Turkey

**Keywords:** Proteins, Molecular medicine, Protein design, Angiogenesis, Immunotherapy

## Abstract

Single chain antibody fragments (scFvs) are favored in diagnostic and therapeutic fields thanks to their small size and the availability of various engineering approaches. Linker between variable heavy (V_H_) and light (V_L_) chains of scFv covalently links these domains and it can affect scFv’s bio-physical/chemical properties and in vivo activity. Thus, scFv linker design is important for a successful scFv construction, and flexible linkers are preferred for a proper pairing of V_H_–V_L_. The flexibility of the linker is determined by length and sequence content and glycine-serine (GS) linkers are commonly preferred for scFvs based on their highly flexible profiles. Despite the advantage of this provided flexibility, GS linkers carry repeated sequences which can cause problems for PCR-based engineering approaches and immunogenicity. Here, two different linkers, a repetitive GS linker and an alternative non-repetitive linker with similar flexibility but lower immunogenicity are employed to generate anti-Vascular Endothelial Growth Factor scFvs derived from bevacizumab. Our findings highlight a better in vitro profile of the non-repetitive linker such as a higher monomer ratio, higher thermal stability while there was no significant difference in in vivo efficacy in a zebrafish embryonic angiogenesis model. This is the first study to compare in vivo efficacy of scFvs with different linkers in a zebrafish model.

## Introduction

Monoclonal antibodies (mAbs) and antibody fragments are used in a variety of therapeutic and diagnostic applications^1^. Due to its small size and availability of various protein engineering techniques, single chain variable fragment (scFv) is one of the most utilized antibody fragments. scFv is composed of variable domains of heavy (V_H_) and light (V_L_) chains of mAbs that are covalently linked together by a flexible peptide linker. Linker nature is key to forming a proper V_H_–V_L_ antigen-binding interface affecting scFv function. scFv linker is critical due to its effects on both its in vitro and in vivo properties^2,3^. Therefore, peptide linker design is key for a successful scFv construction^4,5^.

The length and sequence content of the linker are two features that can affect expression level, folding, oligomeric state, affinity/specificity, stability, and in vivo activity of scFvs^5–8^. Natural and synthetic linkers are being studied for fusion proteins and they are broadly divided into three groups: (i) flexible, (ii) rigid, and (iii) cleavable^7,9^. Glycine-serine (GS) repeat is the most common linker sequence in scFv design mostly due to their flexible nature. GS linkers are utilized in some of the very first scFv fragments^10^. Different lengths and combinations of GS linkers are tested for scFv fragments, the most common ones are (G_3_S)_n_ and (G_4_S)_n_ motifs^3,11–13^. The length of the linker can be optimized from 5 to 35 amino acids to develop improved scFvs for various applications^5,14^. If the scFv linker length is longer than 12 residues, covalently linked V_H_ and V_L_ form a functional scFv and they are supposed to be highly monomeric. scFvs with shorter linkers (< 12 a.a.) tend to form multimers by combining with other scFv molecule(s)^15,16^.

Although GS linkers provide the flexibility which is desired for proper scFv folding and structure, they are repetitive which can cause problems related to PCR-based engineering strategies^17^ and immunogenicity^18^. Therefore, alternative non-repetitive linkers with comparable flexibility can be employed for improved in vivo properties. In this study, we compared in vitro and in vivo properties of repetitive and non-repetitive linker sequences utilized in an anti-Vascular Endothelial Growth Factor (VEGF) scFv. The role of VEGF is critical as it is a key driver of the sprouting angiogenesis in tumor growth^19^. This has led to the development of anti-VEGF therapeutic approaches and many therapeutic drugs against VEGF are being used^20,21^. This is the first study to compare in vivo efficacy of scFvs with different linkers on a zebrafish model. Although non-repetitive linker showed better in vitro properties, there was no significant difference in in vivo efficacy. More importantly, both of our designed scFvs which are derived from the bevacizumab sequence showed better in vivo effects than bevacizumab itself.

## Results

**Figure 1:**
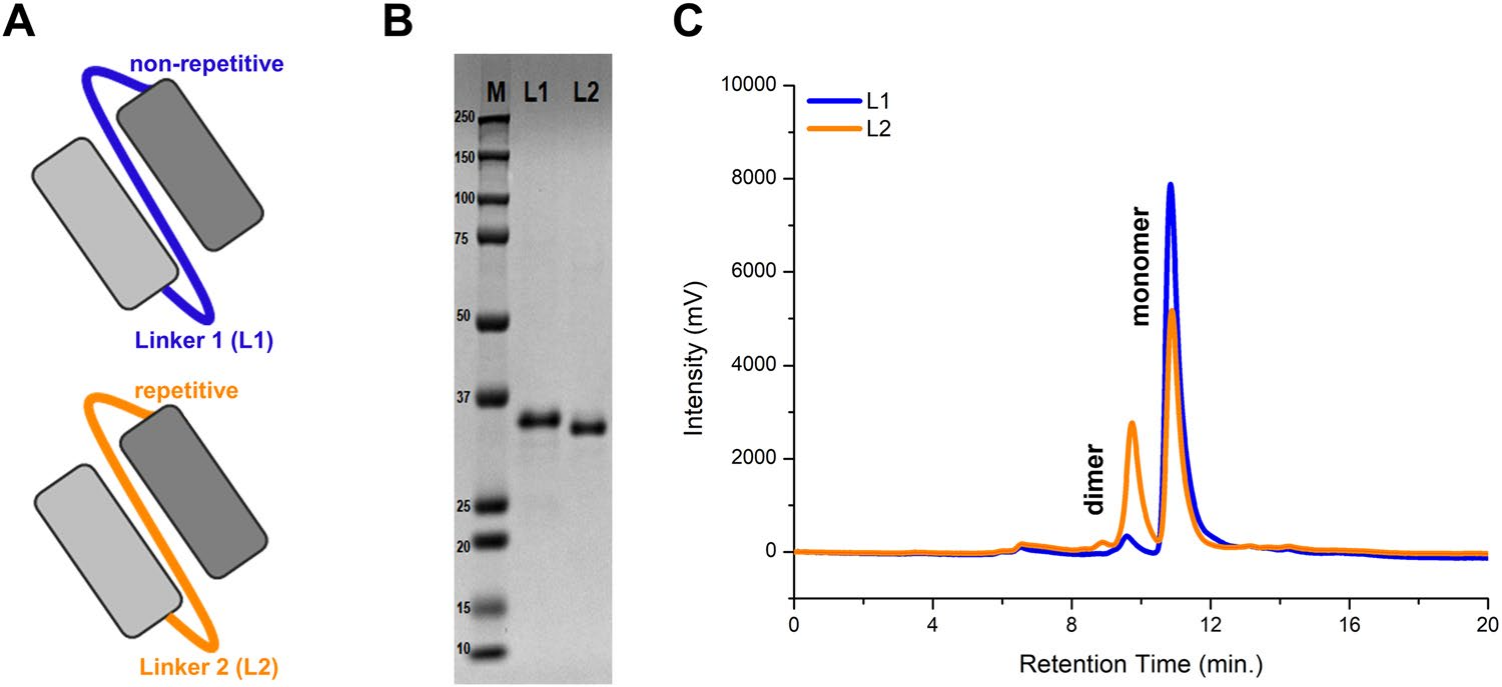
(**A**) Schematic representation of designed L1 and L2 scFvs. (**B**) SDS-PAGE analysis of purified L1 and L2. Full image of the SDS-PAGE gel is provided in Supplementary Figure 1. (**C**) SEC chromatograms of L1 (blue) and L2 (orange) from SE-HPLC analysis.

Two different linker sequences for the same anti-VEGF scFv sequence were used. Both linkers have long lengths for proper structural positioning of linked V_H_ and V_L_ to form monomeric scFv. While one scFv has a non-repetitive linker (L1, SPNSASHSGSAPQTSSAPGSQ), the other has a repetitive linker (L2, (G_3_S)_4_) (Fig. 1A). First, we performed in silico analysis to determine the flexibility of the linkers and it showed that they have very similar flexibilities (Table 1). Immunogenicity propensities were also investigated by a specific IEDB tool^22^. A higher score implies a bigger probability of eliciting an immune response. L2, scFv with a repetitive linker, showed a significantly higher immunogenicity score than L1. Because immunogenicity is undesired in therapeutic interventions, this particular non-repetitive linker might be advantageous compared to the most common GS linker.

**Table 1:** Properties of L1 and L2 variants.

	Flexibility score	Immunogenicity score	MW (kDa)	Monomer/dimer ratio (monomer %)	% Insoluble aggregates*
L1	0.47	−1.09	32.8	95.7 ± 0.7	88.5 ± 2.8
L2	0.53	−0.06	31.9	66.5 ± 3.2	90.2 ± 4.3

*Percent insoluble aggregation was calculated by subtracting soluble protein concentration from total concentration after thermal (60 °C) and mechanical (220 rpm) stress for 4 h. The average of 3 different samples was used.

L1 and L2 were expressed in *E.coli* and purified from the supernatant by Protein L chromatography with high purity. The molecular weight difference of ~ 1 kDa due to the linker sequence difference can be distinguished in SDS-PAGE (Fig. 1B). The effect of the linkers on the oligomeric state of the scFvs was investigated using size-exclusion chromatography (SEC). Retention times of dimeric and monomeric forms were identified using molecular weight protein standards. Percentages of dimeric and monomeric forms in solution were determined using areas under the peaks. According to the results, there is a significant difference in monomer/dimer ratios (Table 1, Fig. 1C). L1 has > 95% monomer while L2 has 66.5% monomer. A higher monomer ratio is more desired because it is known to be thermodynamically more stable^23^.

**Figure 2:**
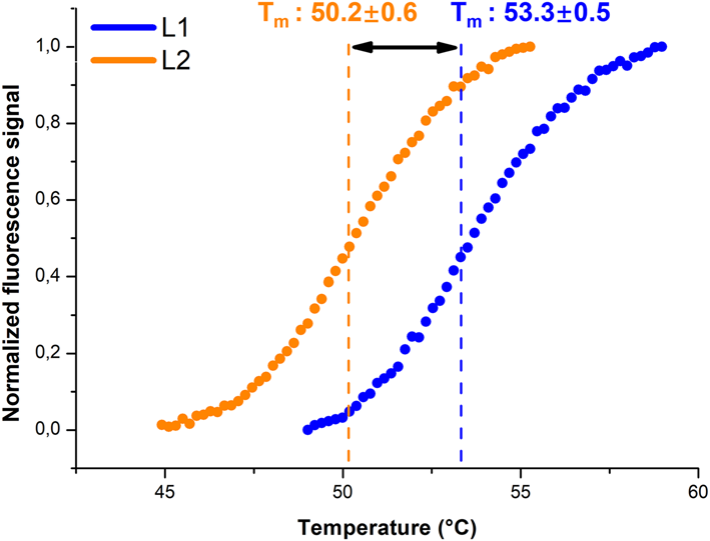
Thermal melting temperatures of L1 and L2. Transition mid-points (T_m_ values) from fluorescent thermal melt assays were calculated by Hill equation fit. The assay was repeated 3 times.

Aggregation propensities of L1 and L2 were examined under stress conditions. Aggregation of the proteins was induced by both mechanical (shaking) and thermal (heating) stress and soluble fractions of the proteins after precipitation of aggregate forms were measured. There was no significant difference in aggregation profiles of L1 and L2 (Table 1). Thermal stabilities of L1 and L2 were determined by thermal denaturation assay. A fluorescent dye that binds to hydrophobic regions was used to monitor protein unfolding under thermal stress. Melting temperature (T_m_) at which the half percentage of protein is unfolded was calculated. L1 was more stable compared to L2 indicating that non-repetitive linker provided more thermal stability to the scFv (Fig. 2).

**Figure 3:**
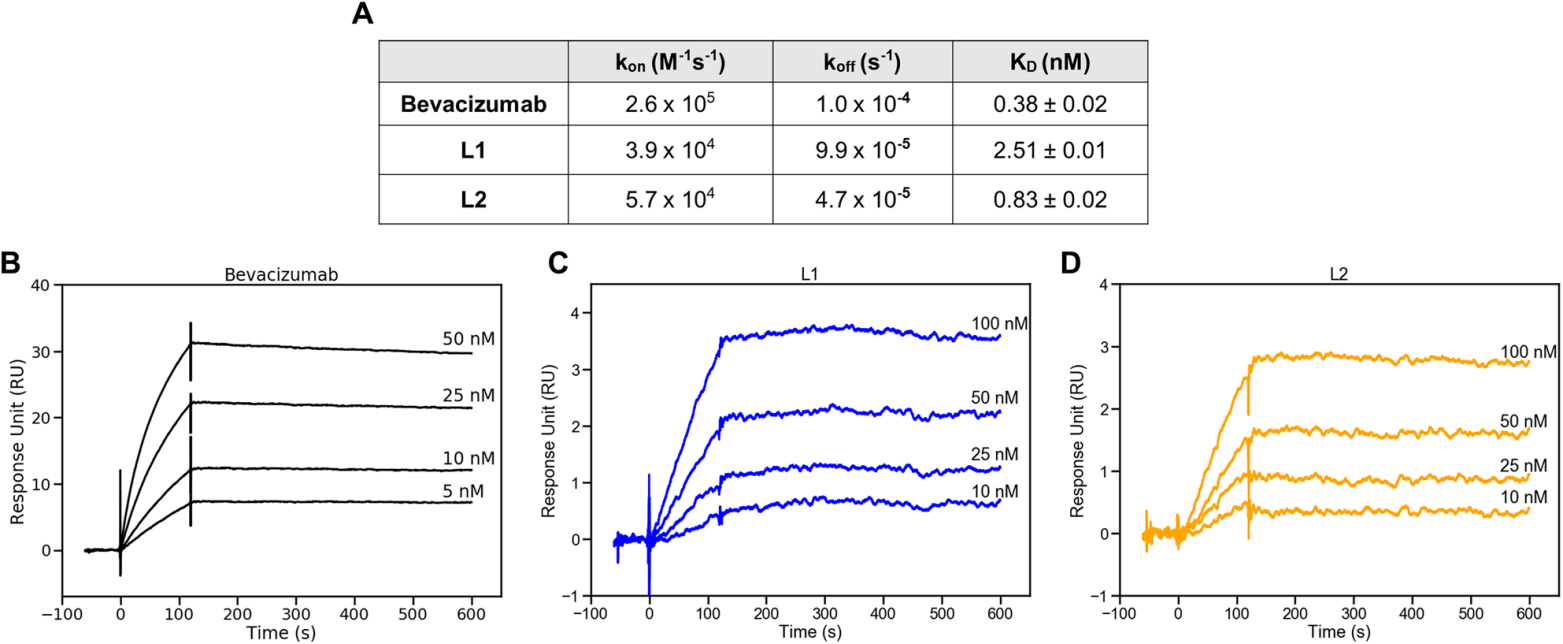
Binding kinetics of Bevacizumab, L1 and L2 fragments to their antigen, VEGF. (**A**) Obtained kinetic parameters. Sensogram overlays for (**B**) bevacizumab (**C**) L1 and (**D**) L2. Antibody concentrations are expressed on corresponding curves. Analyses were repeated 2 times.

Binding kinetics of L1, L2, and bevacizumab (IgG) were analyzed based on their bindings to their ligand, VEGF. Surface Plasmon Resonance (SPR) was used and corresponding association (k_on_), dissociation (k_off_) constants, and binding affinity (K_D_) were obtained (Fig. 3). A comparison of the kinetic parameters is listed in Fig. 3A. Binding affinities were determined as 0.38 nM, 2.51 nM and 0.83 nM for Bevacizumab, L1 and L2, respectively. Both scFvs have close affinities to Bevacizumab. L2 showed approximately threefold better binding to VEGF compared to L1. The significant difference in oligomeric states of scFvs caused by different linkers might be effective on this difference in binding characteristics.

In vivo﻿ anti-angiogenesis activities of L1 and L2 were tested with the zebrafish subintestinal vessel (SIV) assay, using *fli1:EGFP* transgenic zebrafish line which drives GFP expression in endothelial cells^24^. L1, L2, Bevacizumab, or PBS was injected into the yolk of embryos at 48–52 h post fertilization (2dpf) at the initiation phase of SIV formation^25^. The SIV development is driven by VEGF in zebrafish the anti-VEGF agents and antibodies were shown to inhibit SIV development in zebrafish^26^. Antibodies L1 (55 μM), L2 (55 μM) and bevacizumab (27.5 μM) were injected into the yolk of 2 dpf (48–52 hpf) zebrafish embryo, the effect was quantified the next day by analyzing the SIV area. Half concentration for bevacizumab was used because bevacizumab is divalent (1:2 antibody: VEGF binding) and scFvs are monovalent (1:1).

**Figure 4:**
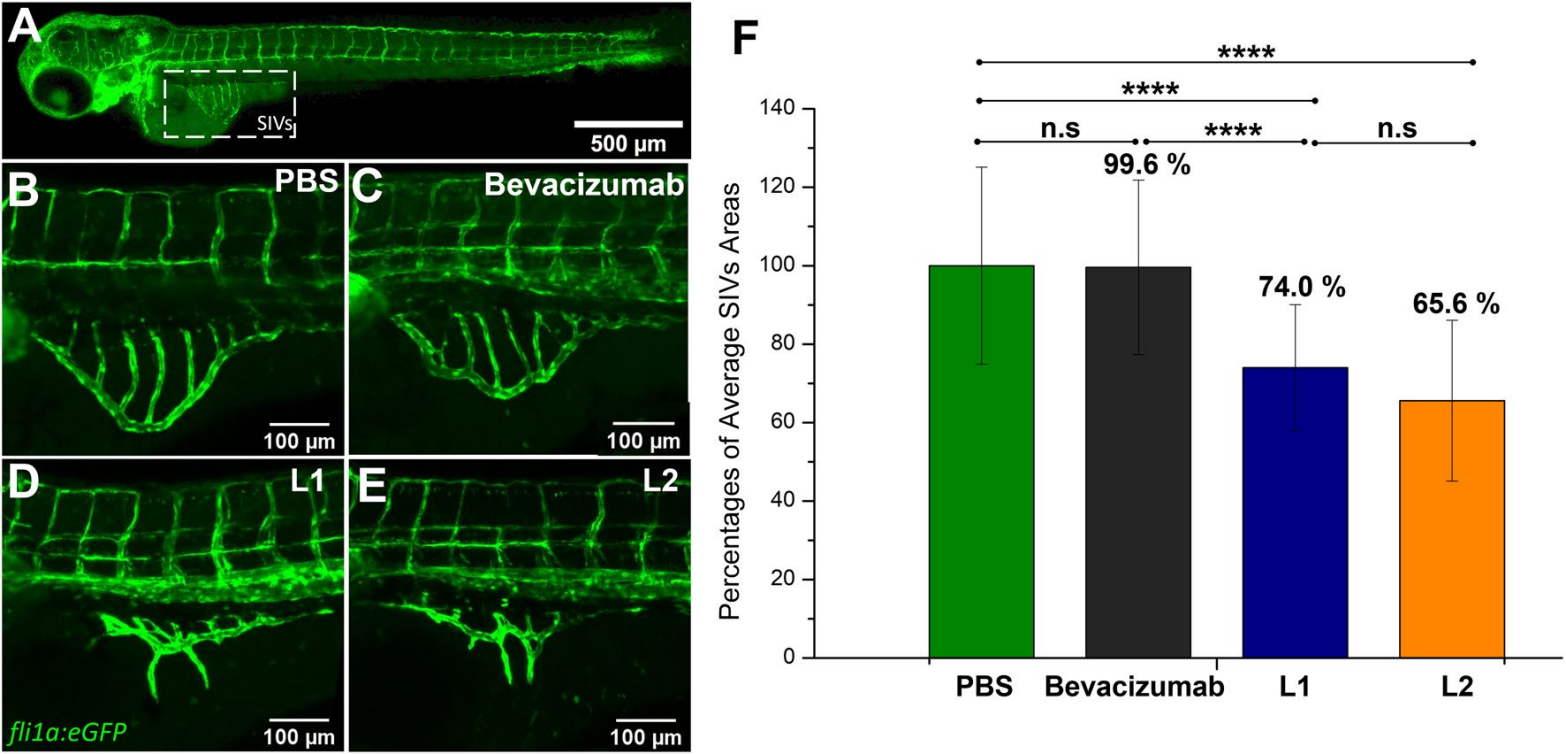
In vivo angiogenesis inhibition by L1, L2 and bevacizumab. (**A**) Lateral view of subintestinal vessels (SIVs) in *fli1:EGFP* transgenic zebrafish larvae at 3 dpf. (**B**) PBS (negative control) (**C**) 27.5 μM bevacizumab (**D**) 55 μM L1, (**E**) 55 μM L2 were injected into yolk of 2 dpf *fli1:EGFP* transgenic zebrafish embryos. At 3 dpf, zebrafish SIVs were imaged by confocal microscopy. (**F**) Percentages of average SIV areas were quantified. The outcomes are expressed as AVG ± SD. n_PBS_ = 25, n_bevacizumab_ = 24, n_L1_ = 24, n_L2_ = 23. Statistical analysis was performed using one-tail t-test. Statistical results: n.s. p > 0.05, ****p < 0.0001.

SIVs of embryos treated with 1X PBS were ordered and intact as expected, whereas L1 and L2 inhibited SIV development in the majority of embryos (Fig. 4). To quantify the inhibition, the SIV area in each embryo was measured and the relative mean area with respect to the control group was depicted as percentages (Fig. 4F). When compared to 1X PBS control, L1 and L2 induced a decrease in SIV areas by 26% and 34.4%, respectively (Fig. 4F). However, bevacizumab did not show a significant effect at 27.5 μM. When a higher dose (55 μM) of bevacizumab was injected, a 35.6% decrease in SIV area was observed, similar to that of L2 (data not shown).

## Discussion

Linker contents of scFv are known to affect their in vitro and in vivo properties, so linker design is critical for the developability of an scFv. (G_n_S)_n_ linkers are the most common linkers due to their shown flexibility. In a comparative study performed by Vihinen, *et. al.*, amino acids with bulky side chains such as lysine, and aspartate also have higher flexibility^27^. The repetitive sequence of the GS linker can cause issues during the introduction of random mutations via mutagenic DNA shuffling or during PCR-based assembly of amplified variable domains, which may result in undesired length variants due to improper annealing of homologous sequences of these repetitive sequences^28,29^. There is also a high possibility of having a higher immunogenic effect of repetitive linkers due to their O-glycosylation patterns^30^. Plückthun, et al*.* addressed these issues by designing and selecting non-repetitive linker sequences without disrupting desired properties such as proper folding, solubility, and binding by using the selectively infective phage technology^17^. Three non-repetitive linkers were chosen and further characterized in that work and shown to be equivalent to the original scFv fragment with the (G_4_S)_3_ linker.

Although linker design and engineering are very important in scFv construction, alternative approaches to GS linkers are very limited in the literature. Klement, et al*.* screened natural and artificial linkers for their cytotoxic antibody fragment^31^. They showed that structural stability and functionality were significantly affected by linker content and a natural linker composed of IgG3 upper hinge region had the highest functionality and stability. In one study, three different GS-bearing linkers with neutral, positive, and negative charges were employed and no significant difference was observed for their biodistribution patterns in tumor-bearing mice^32^. In another study, GS and only glycine-containing linkers varying from 1 to 25 amino acids were screened and GS linkers showed better biophysical characteristics and pharmacokinetic properties^14^.

Here, we employed two distinct types of linkers to design an anti-VEGF scFv antibody derived from bevacizumab^33^. One is the most preferred flexible and repetitive GS linker ((G_3_S)_4_, L2), and the other is a non-repetitive linker of "SPNSASHSGSAPQTSSAPGSQ" (L1)^17^. While L2 is a simple linker with repeated glycine and serine residues, it might lead to reduced stability and/or higher immunogenicity which is undesired in therapeutic antibody development. First, developed scFvs were compared to each other in terms of their immunogenicity and flexibility scores using in silico tools. Although their flexibilities were similar, the non-repetitive linker was predicted to be less immunogenic. Both L1 and L2 have linkers with sufficient length and flexibility which should allow both scFv antibodies to be predominantly monomeric structure^34^. Shorter linkers (less than 15 amino acids) enhance oligomerization due to pairing constraints of covalently linked V_H_ and V_L_ domains^35^. Higher-order oligomers of scFvs tend to have decreased overall stability and higher aggregation propensities^14,36^. Thus, both linkers which are longer than 15 amino acids should allow the domains to pair in the proper orientation for dominantly monomeric forms. First, we analyzed oligomeric states of L1 and L2 by SE-HPLC. As expected, both scFvs were dominantly monomeric. However, scFv with the non-repetitive linker had a significantly higher monomeric form which is desired (95.7% and 66.5% for L1 and L2, respectively). By using in vitro assays, we also compared the thermal stability, aggregation resistance, and affinity characteristics of these two scFvs. They did not show a significant difference in terms of their aggregation tendencies after thermal and mechanical stress. While L1 had significantly higher thermal stability, it had ~ 3 × less affinity to VEGF. It is important to note that VEGF affinities of L1 and L2 were close to bevacizumab which are all within the therapeutic range. (Fig. 3). This shows that the binding affinity of engineered scFvs is usually preserved upon exclusion of constant regions^37^. These in vitro results show that the structural stability of the scFv with the non-repetitive linker is better with a slight decrease in binding functionality.

In vivo efficacy of these scFv antibodies were compared with bevacizumab using the zebrafish (*Danio rerio*) SIV assay. According to our knowledge, this is the first study investigating the linker effect on scFv antibodies by using a zebrafish model. Zebrafish SIV development is induced by VEGF, and previous studies showed that human anti-VEGF antibodies can inhibit this process^38^. Due to the transparency of the zebrafish and, the robustness of the embryo and larvae for micromanipulation, zebrafish is an advantageous model for in vivo testing of anti-VEGF antibodies^39,40^. In this study, we tested the in vivo efficacies of L1 and L2 scFvs and compared them to that of bevacizumab. There was no systemic deformation observed after microinjections. Both L1 and L2 scFvs were found to be more effective than bevacizumab when applied at the same stoichiometric ratio with respect to VEGF binding. Although bevacizumab is divalent and can bind to VEGF at a 1:2 ratio, and scFvs are monovalent and bind to VEGF at a 1:1 ratio, the half concentration of bevacizumab was not as effective as scFvs. When 55 μM of L1 or L2 were injected, SIV development was inhibited which resulted in an irregular structure and a reduction in the area of SIVs. According to statistical comparisons with the control group (1X PBS), 26% and 34.4% reductions were observed for L1 and L2, respectively. However, bevacizumab as positive control did not show a significant effect at 27.5 μM. When the bevacizumab concentration was increased 2 × to 55 μM, it caused a 35% reduction in the SIV area, a comparable activity to our scFvs. Considering that scFv is a fragment of bevacizumab, it is expected to be as effective as or more effective than bevacizumab. Although bevacizumab had similar in vivo efficacy when used as 2 × more stoichiometric ratio concentration, it can be concluded that bevacizumab was less effective than designed scFvs. It is known that smaller antibody fragments have better tissue penetration^41–43^, so better efficacy of our designed scFvs in the zebrafish model can be explained based on their fivefold smaller sizes (~ 30 kDa) compared to parent bevacizumab (~ 150 kDa). Also, the biodistribution coefficient (estimation of tissue distribution based on plasma concentration) of smaller size antibody fragments was found to be higher which would increase drug efficacy in targeted tissue^44^. Effective scFvs can be derived from commonly used IgGs with several protein engineering techniques^10,45–49^. Overall, this study shows that scFvs can be potentially used for diagnostic and therapeutic approaches and linker design is important for some of their key developability characteristics.

## Methods

### Genes and protein expression

Anti-VEGF scFv sequence was derived from bevacizumab. Bevacizumab sequence was extracted from the DrugBank database with the accession number DB00112^50^. Antibody residues were numbered according to the Kabat numbering system. Variable domain residues were determined via analysis of PDB structure of bevacizumab, 1BJ1^51^*.* C-terminal of the variable domains sequences are determined after the last β-sheet and the first 2 residues of the linker region for the heavy and light chain*.* These determined heavy (V_H_) and light (V_L_) chain sequences were linked via either a non-repetitive or repetitive linker. scFv with non-repetitive linker (L1) and repetitive linker (L2) has linker sequences of “SPNSASHSGSAPQTSSAPGSQ” and (G_3_S)_4_, respectively. L1 sequence was selected from the study of Hennecke, et al*.* with one amino acid difference from the reference linker (glutamine instead of asparagine at position 13) which gave lower immunogenicity and higher flexibility score^17^. scFv variants with a leader sequence (PelB), FLAG-tag and penta histidine-tag were transformed into *E.coli strain BL21 (DE3) pLysS* (Thermo Fisher) with pET17-b (GenScript) expression plasmid. Transformant cells were grown on LB-agar plate containing 100 μg/mL ampicillin and 25 μg/mL chloramphenicol. Single colonies were inoculated in LB broth containing 100 μg/mL ampicillin and 25 μg/mL chloramphenicol and grown overnight at 225 rpm, 37 °C as inoculum. These cells were inoculated into 300 mL autoinduction-media and incubated at 18 °C, 250 rpm for 48 h^52^.

### Protein purification

Cultures were centrifuged at 6500xg at 4 °C (Avanti, Beckman Coulter). Protein-containing supernatant was incubated with His-Pur Ni-NTA resin (Thermo Fisher) for 2 h at 4 °C, mixing gently. The mixture was loaded onto a 10 ml vacuum column (Thermo Fisher) and purified according to the manual’s protocol. 1X phosphate-buffered saline (PBS) with 25 mM imidazole, pH 7.4 and PBS with 500 mM imidazole, pH 7.4 were used as a wash and elution buffers, respectively. Purified proteins were buffer-exchanged^53^ into PBS (pH 7.4) through membrane filtration (Amicon® Ultra-4 Centrifugal Filter Units, MWCO 10 kDa, Merck). Protein samples were then loaded onto the HiTrap™ Protein L column (GE Healthcare) to achieve better purity (> 95%). Protein purities were confirmed by sodium dodecyl sulfate–polyacrylamide gel electrophoresis (SDS-PAGE) analysis. Precision Plus Protein™ Dual Color standard was used as a marker (Bio-Rad). Protein concentration was determined by NanoDrop 2000 (absorbance at 280 nm).

### Computational tools

#### Flexibility analysis

The average flexibility of non-repetitive and repetitive linker sequences are calculated according to the average flexibility index of amino acids^54,55^. Flexibility value calculations for L1 and L2 were done based on their amino acid composition and length.

#### Immunogenicity predictions

The risk of immunogenicity of L1 and L2 was predicted using an online tool from IEDB T Cell Epitopes Immunogenicity Prediction (http://tools.immuneepitope.org/main/)^22^.

### Size-exclusion chromatography (SE-HPLC)

A Shimadzu Prominence HPLC System with UV–VIS detector (Kyoto, Japan), and an analytical TSK-gel SuperSW3000 column (4.6 mm ID x 30 cm, 4 mm) (Tosoh Bioscience, Tosoh, USA) were used to perform SEC. Running buffer was 0.1 M phosphate buffer, and 0.1 M potassium sulfate (pH 6.7). scFv samples were prepared at 0.1 mg/mL and injected into the column at 25 °C with a flow rate of 0.3 mL/min over 20 min. The absorbance values were monitored at 280 nm. Thyroglobulin (M_r_ = 669,000), IgG (M_r_ = 150,000), BSA (M_r_ = 66,400), myoglobin (M_r_ = 17,000), and uracil (M_r_ = 112) were used as molecular weight standards to verify the retention times of the scFv samples.

### Aggregation analysis

Protein aliquots (0.5 mg/mL, 25 μL) were incubated at 60 °C, 220 rpm in a heat block to provide both thermal and mechanical stress to seed aggregation. At different time intervals (0–420 min), protein aliquots were taken and centrifuged at 17,000xg at 4 °C for 10 min to pellet aggregated part. Protein concentrations of soluble fractions were measured using NanoDrop 2000 (absorbance at 280 nm).

### Thermal denaturation assay

Thermal unfolding profiles of purified scFv proteins were determined with SYPRO Orange dye (Sigma, S5692) via ABI 7500 Fast RT-PCR (25–99 °C with 0.05% ramp rate). Optimum concentrations of dye and protein were 2 × and 2 μM, respectively. Transition mid-points (T_m_ values) from the thermogram data were calculated using the Hill1 equation fit using the Origin 8.5 software.

### Surface plasmon resonance (SPR)

Affinity measurements were performed using a Biacore T200 instrument (Biacore Inc., Piscataway, NJ). All experiments were performed in HBS-EP buffer, pH 7.4. 1000 nM VEGF protein was immobilized onto a CM4 chip (Cytiva) at a flow rate of 10 µl/min for ∼1 min. A series of solutions ranging from 10 to 100 nM scFvs and 10 to 50 nM bevacizumab were subsequently injected at a flow rate of 30 µl/min onto the VEGF-coated surface. Data were corrected by double-referencing against a control flow cell containing no VEGF and against the flow cell with buffer injection. Sensogram curves were analyzed using the BiaEval 3.0 manufacturer's software. The dissociation constant (*K*_D_), association rate constant (k_on_) and dissociation rate constant (k_off_) values were calculated by fitting the kinetic association and dissociation curves to a 1:1 binding model.

### Zebrafish experiments

Zebrafish used in this study were provided by the Zebrafish Facility in Izmir Biomedicine and Genome Center. All animal procedures were approved by the IBG Local Ethics Committe for Animal Experimentation (IBG-HADYEK) with protocol no 2020-013.

Adult zebrafish were maintained under standard conditions at 28 °C on a 14/10-h light/dark cycle, at the Izmir Biomedicine and Genome Center Zebrafish Facility. Transgenic *fli1:EGFP *line^24^ was crossed to wild type AB to obtain the embryos, which were collected within 30 min of fertilization, incubated in E3 embryo medium (5 mM NaCl, 0.17 mM KCl, 0.33 mM CaCl_2_,0.33 mM MgCl_2_, %1 methylene blue) at 28 °C in a dark incubator.

#### Microinjection and fixation

55 μM L1, L2 and 55 or 27.5 μM bevacizumab (in PBS and 0.5% Phenol red) were kept on ice until microinjection. The estimated injection volume was determined by the use of a stage micrometer and 50 nL of L1, L2, and bevacizumab were microinjected into the yolk of each anesthetized zebrafish embryo (0.02% tricaine) at 2 days post fertilization (dpf) (n > 20 embryos per condition). Microinjected larvae were kept at 28 °C and at 1-day post-injection (dpi), development of subintestinal vessels (SIVs) were examined. Larvae were fixed with fresh 4% formaldehyde, washed with PBS, melanocytes were decolorized with pigment discoloration solution (5% of KOH, 30% of H_2_O_2_, PBS (1:1:8)). Samples were kept at 4 °C until imaging.

#### Imaging and quantification

Larvae were mounted laterally in 1% low melting agarose in imaging dish (D35-14–1.5 N Cell Vis, USA), imaged with Zeiss LSM880 confocal microscope. SIV area was measured with ImageJ polygon tool, statistical analysis was performed with Excel using the one-tail t-test with a confidence interval of 95%. Statistical significances were represented with stars of p-values: non-significant (ns) > 0.05, *p ≤ 0.05, **p ≤ 0.01, ***p ≤ 0.001, ****p ≤ 0.0001. The outcomes are expressed as average (AVG) ± standard error of the mean (SEM).

## Supplementary Information

Supplementary Information.
