# Supplementary material for: Effect of non-repetitive linker on in vitro and in vivo properties of an anti-VEGF scFv
Source: Sci Rep. 2022 Mar 31;12:5449. doi: 10.1038/s41598-022-09324-4 (PMC8971466; doi:10.1038/s41598-022-09324-4)
Supplement: Supplementary file 1 — Supplementary Information. [file 41598_2022_9324_MOESM1_ESM.docx]

**Supplementary Figure 1 :** Full-image of the SDS-PAGE gel.The part of L1 and L2 samples was cropped from the original image and its mirror version is used for Figure 1.B. Cropped image was highlighted with a black box.
